# The Gramine Route to Pyrido[4,3-*b*]indol-3-ones – Identification of a New Cytotoxic Lead

**DOI:** 10.3797/scipharm.1011-11

**Published:** 2010-12-18

**Authors:** Uwe Wollein, Franz Bracher

**Affiliations:** Department of Pharmacy – Center for Drug Research, Ludwig-Maximilians University, Butenandtstr. 5–13, 81377 Munich, Germany

**Keywords:** gamma-Carboline, Gramine, Lactam, Cytotoxic activity, Antifungal activity

## Abstract

A novel approach to 3-oxo-γ-carbolines was worked out starting from methyl indol-2-ylacetate via a gramine derivative. After quaternization, ammonia and 4-methoxybenzylamine could be inserted giving appropriate 3-oxo-γ-carbolines. Condensation with 2-chlorobenzaldehyde under microwave irradiation gave a 4-(2-chlorobenzyl)-3-oxo-γ-carboline. *N*-methylation lead to a product with very promising antifungal and cytotoxic activities.

## Introduction

Natural products are a important source for innovative drugs, and a very large number of marketed drugs consist of natural products or derivatives thereof [1]. Among the bioactive natural products alkaloids play a major role, and β-carboline alkaloids (pyrido[3,4-*b*]indoles) have attracted great interest due to their broad spectrum of biological activities [2].

In the course of our recent investigations on bioactive compounds from natural sources, we had a strong focus on 1-oxo-β-carboline alkaloids and synthetic analogues thereof. So we worked out efficient strategies for the construction of 1-oxo-β-carbolines [3–6] and hybrids between 1-oxo-β-carbolines and other alkaloids [7].

Among the most fascinating compounds from our recent investigations was the 1-oxo-β-carboline alkaloid bauerine C (**A**) [8], which exhibits significant cytotoxic and antiviral activities. The first total synthesis of this alkaloid was worked out by our group [5], and opens the opportunity for further analysis of structure-activity relationships. Meanwhile, numerous 1-oxo-β-carbolines have been reported to exhibit significant biological activities like inhibition of protein kinases [9–11].

As a useful supplement to the investigations on 1-oxo-β-carbolines mentioned above, we intended to work out a synthetic approach to the isomeric 3-oxo-γ-carbolines (pyrido[4,3-*b*]indol-3-ones, **B**).

**Fig. 1.**
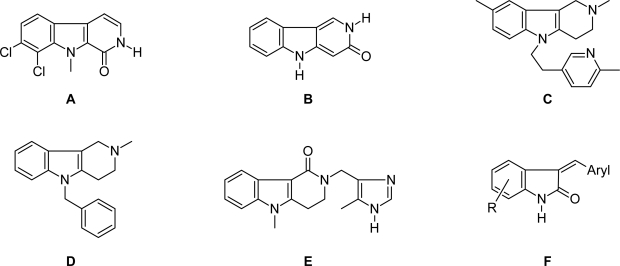
Bauerine C (**A**), 3-oxo-γ-carboline (**B**), latrepirdine (**C**), mebhydrolin (**D**), alosetron (**E**), arylidene oxindoles (**F**)

The γ-carbolines have found only poor application in drug therapy until now, presumably due to their poor synthetic availability. Among the few prominent examples are latrepirdine (former name: dimebolin; **C**), a candidate for treatment of Huntington disease [12], the antihistaminic mebhydrolin (**D**) [13], and the serotonin receptor antagonist alosetron (**E**) [14] (Figure 1). An overview over other biological activities of γ-carbolines is given in reference [15].

Until now only very few approaches to 3-oxo-γ-carbolines have been worked out, and the known strategies only lead to products with additional substituents at C-1 [16] or C-4 [17, 18].

Here we report on a novel approach to 3-oxo-γ-carbolines and further functionalizations, ending up with the discovery of a new cytotoxic lead structure. Homologous tetrahydroazepino[4,5-*b*]indolones [19] have been reported earlier, but no data on biological activities of these products are available.

## Results and Discussion

A retrosynthetic analysis suggested performing the construction of the target 3-oxo-γ-carboline ring system by a final ring closure via lactamization; hence an indole bearing a (derivatized) carboxymethyl group at C-2, and an aminomethyl group at C-3 was selected as precursor.

Methyl indol-2-ylacetate (**1**) is readily available by homolytic alkylation of indole with methyl iodoacetate promoted by hydrogen peroxide and iron(II) sulfate following Baciocchi’s procedure [20]. Subsequent aminomethylation with *N*,*N*-dimethylmethyleneiminium chloride (Eschenmoser’s salt) [21] gave the gramine derivative **2** in good yield. This compound was treated with methyl iodide in tetrahydrofuran to give a quaternary intermediate, which upon treatment with aqueous ammonia gave the target tricyclic lactam **3a** in 51% yield. In this reaction ammonia performs a nucleophilic substitution at the quaternary ammonium group under extrusion of trimethylamine; the intermediate primary amine then undergoes spontaneous lactamization with the ester group. In an analogous manner, the quaternary ammonium salt was reacted with 4-methoxybenzylamine to give the *N*-benzylated lactam **3b** in 34% yield.

Since the methylene group at C-4, next to the lactam carbonyl group in **3a**, should be slightly CH-acidic, we intended to perform some condensation reactions at this position. Upon reaction with aromatic aldehydes α-arylidene lactams should result. The target compounds show similarity to arylidene oxindoles (**F**; Figure 1), which are known as antitumor agents targeting various kinases (e.g. sunitinib [22]), and have very recently been described as sirtuin inhibitors by our group [23]. First attempts at condensation of lactam **3a** with aromatic aldehydes by refluxing the components in ethanol or toluene in the presence of bases (piperidine, pyrrolidine) following Sun’s methodology [24] gave disappointing results; no conversion was observed at all. Finally we were able to perform the Knoevenagel-type condensation of **3a** with 2-chlorobenzaldehyde by using Ding’s protocol [25]. Both starting materials were loaded on a commercial potassium fluoride on aluminium oxide support, and the resulting solid was heated in a microwave reactor without solvent for 8 min (maximum temperature: 150 °C). However, the condensation product obtained in 20% yield did not have the expected arylidenelactam structure **5**, but was identified as the tautomeric 4-(2-chlorobenzyl)-3-oxo-γ-carboline **4**. The structure was confirmed unambiguously by NMR experiments. In a HMBC experiment (which detects ^2^J and ^3^J C-H couplings) the methylene protons showed 6 couplings, in accordance with structure **4**; tautomer **5** would give only 4 couplings in this experiment. NOE experiments showed the physical proximity of the methylene protons and 6’-H at the chlorophenyl residue, as well as the proximity of 1-H and 9-H of the tricyclic core. Unfortunately, analogous condensations with other aromatic aldehydes (benzaldehyde, 4-fluorobenzaldehyde, 3,4,5-trimethoxybenzaldehyde) gave only very poor conversions (<10%), and the purification of the products was difficult.

Finally, we reacted the 4-(2-chlorobenzyl)-3-oxo-γ-carboline **4** with iodomethane and potassium *tert*-butoxide and obtained the 2,5-dimethyl derivative **6**.

The new compounds were submitted to a screening for cytotoxic activity in a standard MTT assay [26] and an agar diffusion assay for detection of antibacterial and antifungal activities. The results are shown in Table 1.

These preliminary screenings clearly indicate that compound **6** has by far the highest biological activities. Its antibacterial activities are significant, but lower than those of the reference antibiotic tetracycline hydrochloride, the antifungal activities against *Candida glabrata* and *Aspergillus niger* are in the same range as those of clotrimazole. And finally, this chlorobenzyl 3-oxo-γ-carboline shows cytotoxic activity comparable to cisplatin. The enormous enhancement of biological activities caused by the introduction of both *N*-methyl groups (**4** → **6**) is noteworthy.

In conclusion, we have worked out a new synthetic approach to the until now poorly investigated class of 3-oxo-γ-carbolines, and have identified compound **6** as a very interesting lead compound for further development. But for being able to prepare a large number of additional analogues for screening and analysis of structure-activity relationships, we will have to work out an optimized protocol for the introduction of benzyl and related moieties at C-4. Work is in progress to find efficient methodologies.

## Experimental

Microwave reactor: CEM Discover. Column chromatography was performed on Kieselgel 60 (230–400 mesh), the solvent ratios are given as v/v. All solvents were dried over appropriate drying agents. Melting points: Büchi Melting point B-540, uncorrected. IR-Spectra: Jasco FT-IR-410. High resolution and mass spectra were obtained on a Hewlett Packard 5989 A and a Jeol M Station 700. ^1^H and ^13^C NMR spectra were recorded on a Jeol JNMR-GSX 400 or a Jeol JNMR-GSX 500, using TMS as internal standard. Elemental analyses were performed on a Varian Vario EL.

### Methyl {3-[(dimethylamino)methyl]-1*H*-indol-2-yl}acetate (2)

To a solution of methyl indol-2-ylacetate (**1**, 1.48 g, 7.82 mmol) in 15 ml dichloromethane was added *N*,*N*-dimethylmethyleneiminium chloride (1.00 g, 10.2 mmol). The mixture was stirred at r.t. for 1 h, and after addition of 30 ml water stirring was continued for 3 min. The mixture was adjusted to pH 10 with 3 N sodium hydroxide solution and extracted with dichloromethane/methanol (95:5; 3 × 20 ml). The combined organic layers were washed with brine (50 ml), dried over magnesium sulfate, and evaporated in vacuo. Purification was performed by chromatography (ethyl acetate, hexane, *N*-ethyl-*N,N*-dimethylamine, 80:20:2) to yield **2** as red crystals (897 mg, 61 %); mp 108 °C. ^1^H NMR (CD_2_Cl_2_): δ = 8.74 (br s, 1 H, NH), 7.60 (d, *J* = 8.0 Hz, 1 H, 4-H), 7.34 (d, *J* = 8.0 Hz, 1 H, 7-H), 7.13 (ddd, *J* = 1.0, 7.0, 8.0 Hz, 1 H, 6-H), 7.06 (ddd, *J* = 1.0, 7.0, 8.0 Hz, 1 H, 5-H), 3.91 (s, 2 H, CH_2_), 3.73 (s, 3 H, OCH_3_), 3.56 (s, 2 H, CH_2_N), 2.22 (s, 6 H, N(CH_3_)_2_). ^13^C NMR (CD_2_Cl_2_): δ = 171.4, 135.9, 129.5, 129.0, 122.0, 119.8, 119.3, 111.0 (2 C), 53.6 (2 C), 52.6, 45.4, 31.9. MS (EI, 70 eV): *m/z* (%) = 246 (12, M^+^), 201 (100), 169 (76), 142 (74). IR (KBr) ν, cm^−1^: 3408, 2862, 1737, 1456, 1206, 1170, 992. Elemental analysis: Calcd. for C_14_H_18_N_2_O_2_: C, 68.27; H, 7.37; N, 11.37; found: C, 68.76; H, 7.41; N, 11.42.

### 1,2,4,5-Tetrahydro-3*H*-pyrido[4,3-*b*]indol-3-one (3a)

To a solution of gramine derivative **2** (1.90 g, 7.71 mmol) in 60 ml tetrahydrofuran was added iodomethane (10.9 g, 77.1 mmol). After stirring at r.t. for 1 h the solvent was removed under reduced pressure, the residue was dissolved in 180 ml of aqueous ammonia solution (30 %). This mixture was stirred at r.t. for 12 h. Then brine (50 ml) was added and the mixture was extracted with dichloromethane/methanol (95:5; 3 × 75 ml). The combined organic layers were evaporated in vacuo. The residue was digested with cold dichloromethane (20 ml), the remaining residue was collected and crystallized from ethyl acetate to give **3a** as a yellow powder (470 mg, 51 %); mp 303 °C. ^1^H NMR (DMSO-*d_6_*): δ = 10.92 (br s, 1 H, 5-H), 8.05 (br s, 1 H, 2-H), 7.40 (d, *J* = 8.0 Hz, 1 H, 9-H), 7.32 (d, *J* = 8.0 Hz, 1 H, 6-H), 7.06 (ddd, *J* = 1.0, 7.1, 8.0 Hz, 1 H, 7-H), 6.98 (ddd, *J* = 1.0, 7.1, 8.0 Hz, 1 H, 8-H), 4.46 (m, 2 H, 1-H), 3.54 (t, *J* = 3.6 Hz, 2 H, 4-H). ^13^C NMR (DMSO-*d_6_*): δ = 167.9, 136.8, 129.9, 124.8, 121.1, 118.8, 117.7, 111.2, 102.7, 40.6, 30.4. MS (EI, 70 eV): *m/z* (%) = 186 (100, M^+^), 143 (86). IR (KBr) ν, cm^−1^: 3187, 3052, 1666, 1629, 1010, 834. Elemental analysis: Calcd. for C_11_H_10_N_2_O: C, 70.95; H, 5.41; N, 15.04; found: C, 70.74; H, 5.29; N, 14.78.

### 2-(4-Methoxybenzyl)-1,2,4,5-tetrahydro-3*H*-pyrido[4,3-*b*]indol-3-one (3b)

To a solution of gramine derivative **2** (2.30 g, 9.33 mmol) in 60 ml tetrahydrofuran was added iodomethane (13.2 g, 93.3 mmol). After stirring at r.t. for 1 h the solvent was removed under reduced pressure, and the residue was dissolved in 50 ml 4-methoxybenzylamine, and the mixture was stirred vigorously at r.t. for 12 h. Then brine (100 ml) was added and the mixture was extracted with dichloromethane/methanol (95:5; 3 × 50 ml). The combined organic layers were washed with 2 N hydrochloric acid (4 × 50 ml), dried over sodium sulfate and evaporated in vacuo. The residue was purified by chromatography (ethyl acetate, hexane, *N*-ethyl-*N,N*-dimethylamine, 80:20:2), followed by crystallization from methanol to yield **3b** as colourless crystals (964 mg, 34 %); mp 183 °C. ^1^H NMR (DMSO-*d_6_*): δ = 10.97 (br s, 1 H, 5-H), 7.34 (d, 1 H, *J* = 8.0 Hz, 9-H), 7.27 (dt, 2 H, *J* = 9.1, 2.5 Hz, 2′-H, 6′-H), 7.06 (ddd, 1 H, *J* = 8.0, 7.1, 1.0 Hz, 7-H), 6.96 (ddd, 1 H, *J* = 8.0, 7.1, 1.0 Hz, 8-H), 6.90 (dt, 2 H, *J* = 9.1, 2.5 Hz, 3′-H, 5′-H), 4.69 (s, 2 H, CH_2_), 4.51 (t, 2 H, *J* = 3.5 Hz, 1-H), 3.75 (t, 2 H, *J* = 3.5 Hz, 4-H), 3.72 (s, 3 H, OCH_3_). ^13^C NMR (DMSO-*d_6_*): δ = 166.0, 158.7, 136.6, 129.6, 129.5, 129.3 (2 C), 124.6, 121.2, 119.0, 117.6, 114.1 (2 C), 111.3, 102.3, 55.2, 49.1, 45.0, 30.9. MS (EI, 70 eV): *m/z* (%) = 306 (48, M^+^), 144 (100). IR (KBr) ν, cm^−1^: 3243, 2927, 2834, 1619, 1511, 1457, 1369, 1326, 1245, 1176, 1033, 744. Elemental analysis: Calcd. for C_19_H_18_N_2_O_2_: C, 74.49; H, 5.92; N, 9.14; found: C, 74.43; H, 5.96; N, 9.01.

### 4-(2-Chlorobenzyl)-2,5-dihydro-3*H*-pyrido[4,3-*b*]indol-3-one (4)

To a solution of the tetrahydropyrido[4,3-*b*]indol-3-one **3a** (200 mg, 1.08 mmol) and 2-chlorobenzaldehyde (151 mg, 1.08 mmol) in 20 ml acetonitrile 1.70 g potassium fluoride on alumimum oxide (about 5.5 mmol fluoride/g; Aldrich) was added, and the suspension was stirred at r.t. for 5 min. Then the solvent was removed in vacuo, and the finely powdered residue placed in a microwave vial and irradiated at an energy impact of 60 W for 8 min. During this time the mixture reached a maximum temperature of 150 °C. After cooling the mixture was extracted with 20 ml acetonitrile and 20 ml dichloromethane/methanol (95:5). The combined organic layers were evaporated and the residue purified by chromatography (chloroform, ethanol, 9:1) to yield **5** as brownish-yellow crystals (67 mg, 20%); mp 216 °C. ^1^H NMR (DMSO-*d_6_*): δ = 10.9 (br s, 1 H, 5-H), 8.29 (s, 1 H, 1-H), 7.90 (d, 1 H, *J* = 7.7 Hz, 9-H), 7.47 (dd, 1 H, *J* = 7.8, 1.5 Hz, 3′-H), 7.25 (t, 1 H, *J* = 7.7 Hz, 7-H), 7.21 (ttt, 1 H, 7.8, 6.3, 1.5 Hz, 4′-H), 7.18 (d, 1 H, *J* = 7.7 Hz, 6-H), 7.14 (ddd, 1 H, *J* = 7.8, 6.3, 1.5 Hz, 5′-H), 7.07 (t, 1 H, *J* = 7.7 Hz, 8-H), 6.79 (dd, 1 H, *J* = 7.8, 1.5 Hz, 6′-H), 3.98 (s, 2 H, CH_2_). ^13^C NMR (DMSO-*d_6_*): δ = 161.8, 149.8, 142.4, 137.2, 133.5, 128.8, 128.2, 127.3, 127.2, 126.8, 125.9, 122.3, 119.8, 119.7, 110.2, 109.6, 99.7, 29.0. MS (EI, 70 eV): *m/z* (%) = 310 (6, M^+^), 308 (14, M^+^), 273 (100). IR (KBr) ν, cm^−1^: 3419, 1661, 1617, 1490, 1466, 1398, 1235, 1048, 868, 743, 693. HRMS: calcd. for C_18_H_13_ClN_2_O: 308.0711, found: 308.0716.

### 4-(2-Chlorobenzyl)-2,5-dimethyl-2,5-dihydro-3*H*-pyrido[4,3-*b*]indol-3-one (6)

A solution of the pyrido[4,3-*b*]indol-3-one **4** (63 mg, 0.21 mmol) and potassium *tert*-butoxide (27 mg, 0.23 mmol) in 5 ml anhydrous dimethyl sulfoxide was warmed at 80 °C with stirring, and after 30 min cooled to r.t. again. Then iodomethane (178 mg, 1.26 mmol) was added and the mixture stirred at 80 °C for 2 h. The mixture was poured on 5 ml aqueous ammonia (10%), and water (50 ml) was added, followed by extraction with ethyl acetate (3 × 40 ml). The combined organic layers were dried over magnesium sulfate and evaporated. The residue was purified by chromatography (ethyl acetate, ethanol, 9:1) to yield **6** as yellow crystals (17 mg, 24%); mp 210 °C. ^1^H NMR (DMSO-*d_6_*): 8.70 (s, 1 H, 1-H), 7.85 (d, 1 H, *J* = 7.8 Hz, 9-H), 7.50 (dd, 1 H, *J* = 7.6, 1.3 Hz, 5′-H), 7.33 (ddd, 1 H, *J* = 7.8, 7.3, 1.0 Hz, 7-H), 7.28 (d, 1 H, *J* = 7.8 Hz, 6-H), 7.23 (ddd, 1 H, *J* = 7.6, 7.5, 1.3 Hz, 4′-H), 7.17 (ddd, 1 H, *J* = 7.8, 7.3, 1.0 Hz, 8-H), 7.14 (ddd, 1 H, *J* = 7.6, 7.5, 1.3 Hz, 3′-H), 6.82 (dd, 1 H, *J* = 7.6, 1.3 Hz, 2′-H), 4.32 (s, 2 H, CH_2_), 3.62 (s, 3 H, N^2^-CH_3_), 3.50 (s, 3 H, N^5^-CH_3_). ^13^C NMR (DMSO-*d_6_*): δ = 161.1, 147.3, 142.6, 137.8, 131.7, 130.0, 128.1, 128.0, 126.7, 126.4, 125.1, 120.7, 119.5, 117.9, 108.1, 108.0, 99.9, 37.1, 29.5, 27.2. MS (EI, 70 eV): *m/z* (%) = 338 (6, M^+^), 336 (20, M^+^), 301 (100). IR (KBr) ν, cm^−1^: 3427, 3054, 2924, 1705, 1665, 1611, 1588, 1560, 1469, 1442, 1279, 1240, 1089, 1048, 747. HRMS: calcd. for C_20_H_17_ClN_2_O: 336.1032, found: 336.1029.

### MTT assay

A solution of the substance in dimethyl sulfoxide (1 μl, concentrations in the range from 10^−9^ to ^−4^ mol/l) was incubated with 99 μl of a suspension of HL 60 cells (9 × 10^5^ cells/ml) in RPMI 1640 medium (PAA Laboratories) with 10% FKS in 96 well plates for 24 h. Then, 10 μl of a solution of MTT (3-(4,5-dimethylthiazol-2-yl)-2,5-diphenyltetrazolium bromide) in PBS (5 mg/ml) were added and the plate was incubated for another 2 h. The cells were quenched with 190 μl dimethyl sulfoxide and after a few min, the plates were evaluated on a Dynatech MRX at a wavelength of 570 nm; the reference wavelength was 630 nm [26]. The experiments were performed in triplicate.

### Agar diffusion assay

The microorganisms listed in Table 1 were cultivated on AC agar (Aldrich), except *Aspergillus niger*, which was cultivated on potato dextrose broth agar (Aldrich). Paper discs (6 mm diameter) were impregnated with 100 μg of each test substance or 50 μg of the reference drugs and placed on the agar. The bacteria media were incubated for 24 h at 32 °C, the fungi media for 48 h at 28 °C. The diameters of the zones of inhibition were measured manually. The experiments were performed in triplicate.

## Authors’ Statement

### Competing Interests

The authors declare no conflict of interest.

## Figures and Tables

**Sch. 1.**
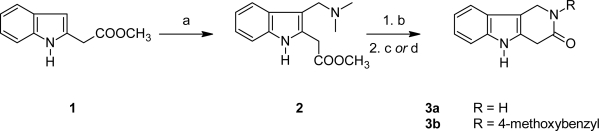
a) *N*,*N*-dimethylmethyleneiminium chloride, dichloromethane, r.t., 1 h (61%); b) iodomethane, tetrahydrofuran, r.t., 1 h; c) conc. ammonia, r.t., 12 h (51% over 2 steps); d) 4-methoxy-benzylamine (neat), r.t., 12 h (34% over 2 steps).

**Sch. 2.**
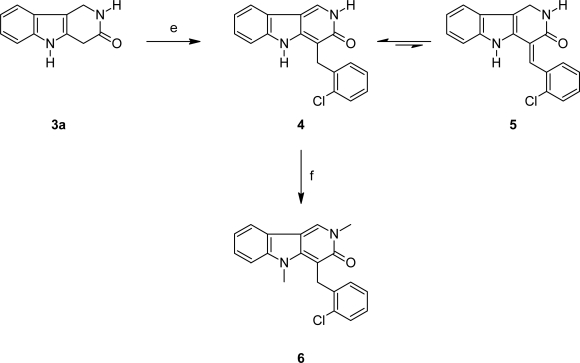
e) 2-chlorobenzaldehyde, potassium fluoride/aluminium oxide, microwave irradiation (20%); f) iodomethane, potassium *tert*-butoxide, dimethyl sulfoxide, 80 °C, 2 h (24%).

**Tab. 1.** Results of the screenings for cytotoxicity (“MTT”; IC_50_ values against HL 60 cells in μM) and for activity against gram-negative bacteria (*Escherichia coli*, “E.coli”.; *Pseudo-monas antimicrobia*, “Pseud”), gram-positive bacteria (*Staphylococcus equorum*, “Staph”; *Streptococcus entericus*, “Strept”), yeasts (*Yarrowia lipolytica*, “Yarr”; *Candida glabrata*, “Cand”), the dermatophyte *Hyphopichia burtonii* (“Hyph”) and the mould *Aspergillus niger* (“Asp”) (diameters of the zones of inhibition in mm). Reference substances: Cisplatin (“CisP”) for the MTT assay; tetracycline hydrochloride (“Tet”) for antibacterial, and clotrimazole (“Clo”) for antifungal activity.

**#**	**MTT**	**E.coli**	**Pseud**	**Staph**	**Strept**	**Yarr**	**Cand**	**Hyph**	**Asp**
**2**	>100	0	0	0	0	0	0	0	0
**3a**	>100	0	10	0	8	0	11	0	10
**3b**	36	0	0	0	0	0	0	0	0
**4**	>100	0	0	0	0	0	0	0	0
**6**	5	17	17	15	8	10	18	0	20
**CisP**	5								
**Tet**		30	32	44	24				
**Clo**						20	18	16	20
